# Understanding of the Site-Specific Microbial Patterns towards Accurate Identification for Patients with Diarrhea-Predominant Irritable Bowel Syndrome

**DOI:** 10.1128/Spectrum.01255-21

**Published:** 2021-12-22

**Authors:** Xue Zhu, Gaichao Hong, Ying Li, Pengshuo Yang, Mingyue Cheng, Lei Zhang, Yuxue Li, Lei Ji, Gangping Li, Chaoyun Chen, Chaofang Zhong, Yu Jin, Min Yang, Hanhua Xiong, Wei Qian, Zhen Ding, Kang Ning, Xiaohua Hou

**Affiliations:** a Key Laboratory of Molecular Biophysics of the Ministry of Education, Hubei Key Laboratory of Bioinformatics and Molecular-imaging, Center of AI Biology, Department of Bioinformatics and Systems Biology, College of Life Science and Technology, Huazhong University of Science and Technology, Wuhan, Hubei, China; b Division of Gastroenterology, Union Hospital, Tongji Medical College, Huazhong University of Science and Technology, Wuhan, Hubei, China; Wayne State University

**Keywords:** IBS-D, multiple intestinal sites, site-specific microbial patterns, microbial biomarkers

## Abstract

Fecal microbial community could not fully represent the intestinal microbial community. However, most studies analyzing diarrhea-dominant irritable bowel syndrome (IBS-D) were mainly based on fecal samples. We aimed to characterize the IBS-D microbial community patterns using samples at multiple intestinal sites. This study recruited 74 IBS-D patients and 20 healthy controls (HC). 22.34%, 8.51%, 14.89%, and 54.26% of them contributed to one, two, three, and four sites: duodenal mucosa (DM), duodenal lumen (DL), rectal mucosa (RM), and rectal lumen (RL) of intestinal samples, respectively. Then 16S rRNA gene analysis was performed on these 283 samples. The result showed that IBS-D microbial communities have specific patterns at each intestinal site differing from that of HC. Across hosts and sites, *Bacillus*, *Burkholderia*, and *Faecalibacterium* were the representative genera in duodenum of IBS-D, duodenum of HC, and rectum of HC, respectively. Samples from mucosa and lumen in rectum were highly distinguishable, regardless of IBS-D and HC. Additionally, IBS-D patients have lower microbial co-abundance network connectivity. Moreover, RM site-specific biomarker: *Bacteroides* used alone or together with *Prevotella* and *Oscillospira* in RM showed outstanding performance in IBS-D diagnosis. Furthermore, *Bacteroides* and *Prevotella* in RM were strongly related to the severity of abdominal pain, abdominal discomfort, and bloating in IBS-D patients. In summary, this study also confirmed fecal microbial community could not fully characterize intestinal microbial communities. Among these site-specific microbial communities, RM microbial community would be more applicable in the diagnosis of IBS-D.

**IMPORTANCE** Microbial community varied from one site to another along the gastrointestinal tract, but current studies about intestinal microbial community in IBS-D were mainly based on fecal samples. Based on 283 intestinal samples collected from DM, DL, RM, and RL of HC and IBS-D, we found different intestinal sites had their site-specific microbial patterns in IBS-D. Notably, RM site-specific microbes *Bacteroides*, *Prevotella*, and *Oscillospira* could be used to discriminate IBS-D from HC accurately. Our findings could help clinicians realize the great potential of the intestinal microbial community in RM for better diagnosis of IBS-D patients.

## INTRODUCTION

Irritable bowel syndrome (IBS) is a functional gastrointestinal disorder (1), with recurrent abdominal pain or discomfort, stool irregularities, and bloating (2), which affected approximately 10% of the population worldwide (3). People with IBS suffer from low quality of life and considerable medication cost, imposing a great burden on society (3). Though the pathophysiology of IBS remains unclear, it has been found that intestinal microbial community dysbiosis contributes to the underlying mechanism of IBS (4).

In recent years, a growing number of researches supported that IBS patients showed altered gut microbial profiles (5), especially in diarrhea-predominant IBS (IBS-D).(6) The intestinal microbial community of these patients would result in the pathogenesis of IBS (7). To be specific, it has already been found that IBS patients exhibited an increased abundance of *Enterobacteriaceae* (8), *Lactobacillaceae* (9), and *Bacteroides* (10), and reduced abundance of *Faecalibacterium* (10) and *Bifidobacterium* (11) compared with healthy individuals.

Current studies on microbial communities of healthy individuals have revealed that the physiological function of different regions in the gastrointestinal (GI) tract would lead to distinct microbial profiles in different sites in GI tract (12–14). Vasapolli et al. identified distinct microbial communities among saliva, upper GI tract, lower GI tract, and fecal samples based on 21 healthy adults (13). Yuan et al. have revealed a location-specific microbial community along the intestinal tract based on mucosal samples collected from 10 adult purpose-bred female olive baboons (14). However, current knowledge about intestinal microbial community in IBS-D was principally based on analysis of fecal microbial community (15, 16). Few studies have described microbial communities combined with different intestinal sites, largely due to the difficulty of collecting samples from these sites, especially for healthy controls (HC).

Currently, IBS can be diagnosed using clinical symptoms according to Rome III (17) or the latest version (Rome IV) criteria (18), which was dependent on clinicians’ knowledge about IBS, so that this was a challenge for the clinicians lacking clinical experience with IBS. Moreover, exhaustive examinations need to be performed to exclude organic diseases before determining the diagnosis of IBS, which leads to substantial medical spending from patients. Additionally, the accurate diagnosis of IBS was challenging with numerous accompanying symptoms and manifestations (19). Furthermore, implementation of intestinal biomarkers in clinical practice is critical for accurate diagnosis of IBS. It was reported that intestinal microbial biomarkers have great potential in identifying inflammatory bowel disease (20), colorectal cancer (21), and autoimmune hepatitis (22). Combining with bioinformatic analysis, we could understand the role of intestinal microbes in IBS-D better, which was beneficial for the diagnosis of IBS-D.

To achieve this, in this study, we collected mucosa and lumen-associated microbial community samples in the duodenum and rectum of IBS-D patients and HC. We determined microbial biomarkers at these sites and produced an efficient classifier to identify IBS-D patients from HC.

## RESULTS

### Overview of sequencing data and clinical symptoms.

The study collected 283 samples from four representative intestinal sites (duodenal mucosa [DM], duodenal lumen [DL], rectal mucosa [RM], and rectal lumen [RL]) of 20 HC and 74 IBS-D patients, resulting in 3,943,124 reads in total and approximately 13,933 reads per sample on average. According to the rarefaction curves (Fig. S1), all curves reached saturation at around 10,000 sequences per sample, suggesting that the sequencing depth was sufficient enough for subsequent analysis. Based on the statistical analysis of clinical symptoms (Table S1), we found that age, gender, and BMI have no significant difference between HC and IBS-D patients. However, IBS-D patients were identified with increased stool consistency, frequency of defecation, and HAD anxiety and depression score compared with HC (*P* < 0.001, Table S1).

### Microbial profile in multi-sites of the intestinal tract.

**FIG 1:**
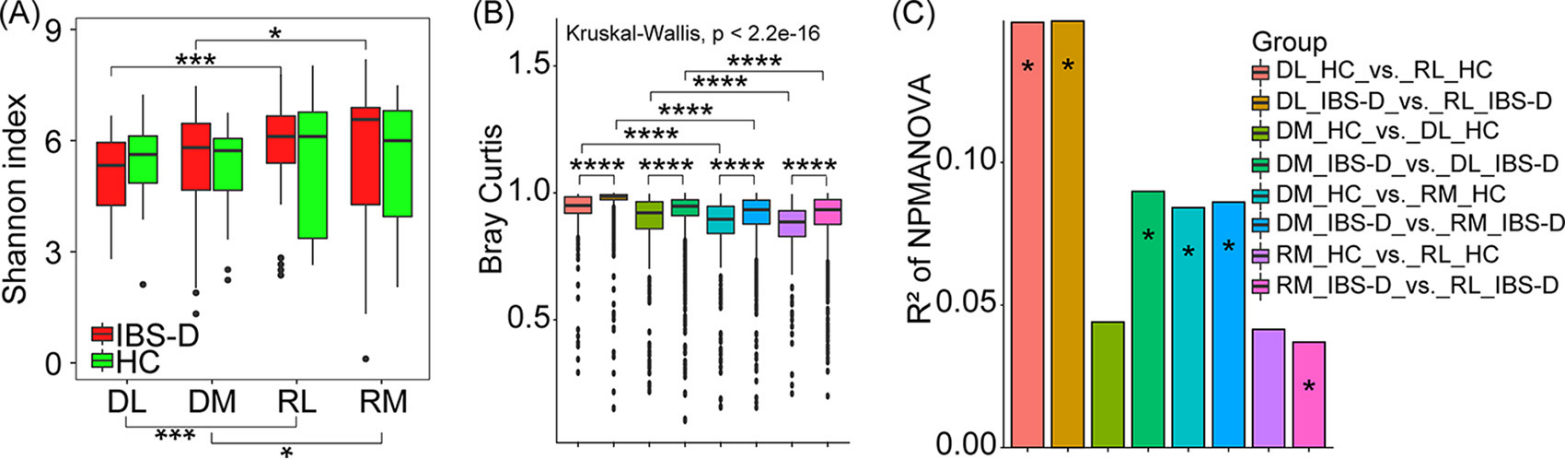
Microbial diversity of duodenum and rectum in HC and IBS-D patients. (A) Comparison of alpha diversity (Shannon index) of microbial communities across intestinal sites in HC and IBS-D patients. (B) Comparison of beta diversity across intestinal sites in HC and IBS-D patients using Bray Curtis distance as the distance measurement. (C) Comparison of beta-diversity of microbial communities across intestinal sites in HC and IBS-D patients based on nonparametric multivariate analysis of variance (NPMANOVA) using Bray Curtis distance as the distance measurement. Kruskal-Wallis was used to detect the global difference, while the Wilcoxon test was used to detect variation across intestinal sites in HC and IBS-D patients by pairwise comparisons based on the microbial composition at genus level. DL_HC, duodenal luminal samples collected from HC; DM_HC, duodenal mucosal samples collected from HC; RL_HC, rectal luminal samples collected from HC; RM_HC, rectal mucosal samples collected from HC; DL_IBS-D, duodenal luminal samples collected from IBS-D patients; DM_IBS-D, duodenal mucosal samples collected from IBS-D patients; RL_IBS-D, rectal luminal samples collected from IBS-D patients; RM_IBS-D, rectal mucosal samples collected from IBS-D patients. *, *P* < 0.1; ***, *P* < 0.01; ****, *P* < 0.001.

**FIG 2:**
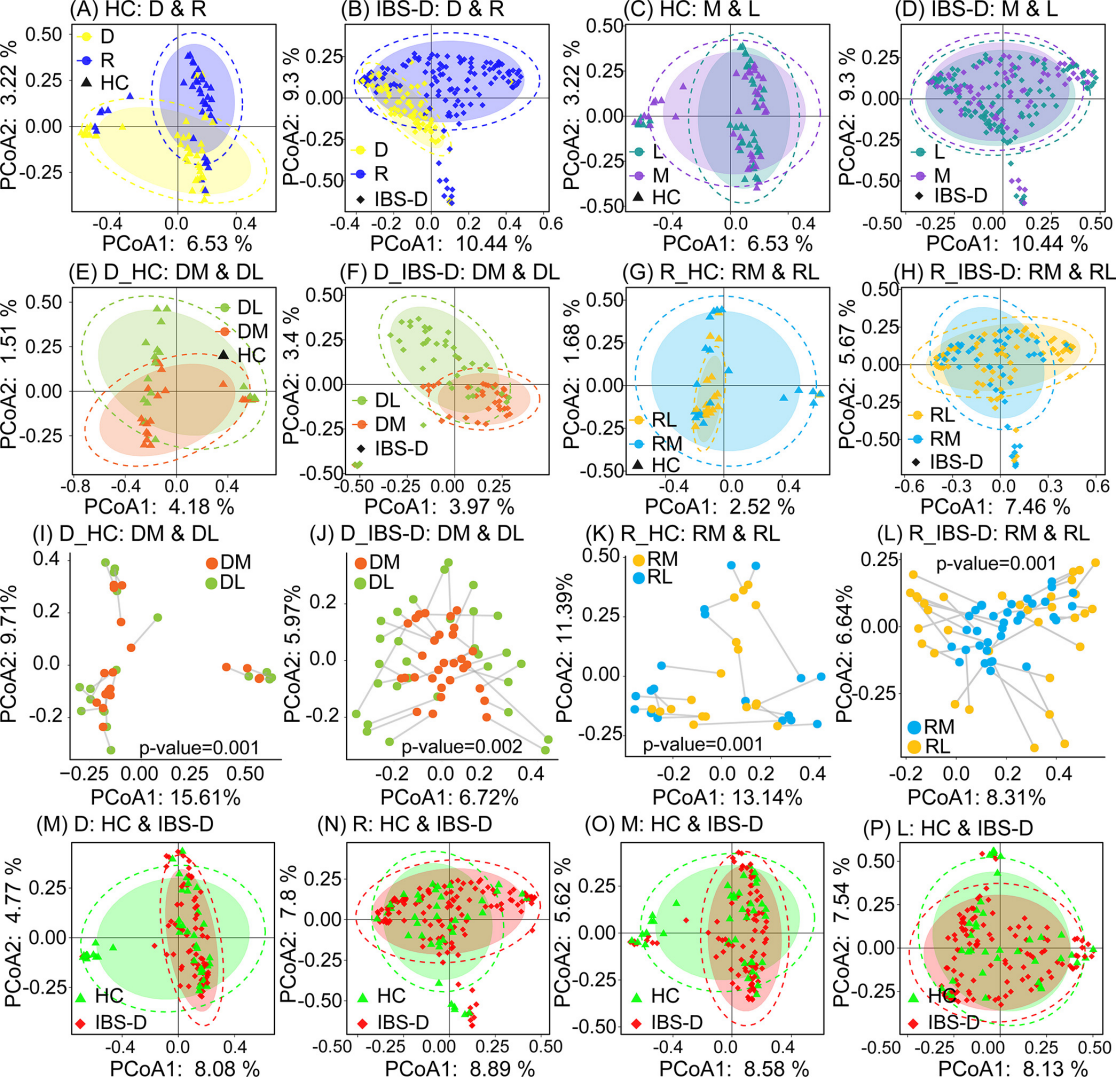
Comparison of microbial communities across intestinal sites in HC and IBS-D. Comparison of microbial communities between duodenum and rectum in all (A) HC and (B) IBS-D samples using PCoA analysis based on Jaccard coefficient as the distance measurement. Comparison of microbial communities between mucosa and lumen in all (C) HC and (D) IBS-D samples. Comparison of microbial communities between DM and DL in (E) HC and (F) IBS-D samples. Comparison of microbial communities between RM and RL in (G) HC samples and (H) IBS-D samples. Procrustes analysis for assessing the similarity of microbial community between DM and DL in (I) HC samples and (J) IBS-D samples, and between RM and RL in (K) HC samples and (L) IBS-D samples, respectively. PCoA analysis for comparison of microbial communities between HC and IBS-D samples within (M) duodenum, (N) rectum, (O) mucosa and (P) lumen. For Procrustes analysis, we utilized the Monte Carlo method with 999 permutations to measure the similarity of the mucosal and luminal microbial communities. The lower *P value* indicated more similarity between two compared microbial communities in Procrustes analysis. All results were based on microbial compositions at genus level. D, all duodenal samples; R, all rectal samples; M, all mucosal samples; L, all luminal samples; DM, duodenal mucosal samples; DL, duodenal luminal samples; RM, rectal mucosal sample; RL, rectal luminal samples; D_HC, duodenal samples collected from HC; D_IBS-D, duodenal samples collected from IBS-D patients; R_HC, rectal samples collected from HC; R_IBS-D, rectal samples collected from IBS-D patients.

IBS-D microbial community was different from that of HC at multiple intestinal sites. We found that alpha diversity of IBS-D microbial community mainly varied across duodenum and rectum (*P* < 0.1), rather than across mucosa and lumen (Fig. 1A). On the contrary, no obvious difference was observed based on alpha diversity of microbial communities across these sites for HC (Fig. 1A). Moreover, IBS-D microbial community was more diverse among all collected intestinal sites compared with HC based on Bray Curtis distance, weighted and unweighted Unifrac distance (*P* < 0.001, Fig. 1B and C and Fig. S2A and B). In overall microbial composition, both IBS-D and HC microbial communities varied from duodenum to rectum, rather than between mucosa and lumen (Fig. 1 and 2, Fig. S3), indicating the disorder of duodenal and rectal microbial community in IBS-D patients. While both PCoA analysis and Procrustes analysis illustrated the mucosa and lumen-associated microbial communities in the duodenum were more distinguishable than those in rectum, regardless of IBS-D and HC (Fig. 2E to L), which again confirmed the fecal microbial community could not be representative of mucosal microbial community (13). These results also warranted the importance of recruiting samples from multiple intestinal sites for analyzing the IBS-D microbial community. Notably, IBS-D microbial community cannot be clearly differentiated from that of HC in PCoA analysis (Fig. 2M to P, Fig. S2C and D and Fig. S4), regardless of using all samples or using site-specific samples, emphasizing the difficulty of identifying IBS-D patients using simple clustering methods.

**FIG 3:**
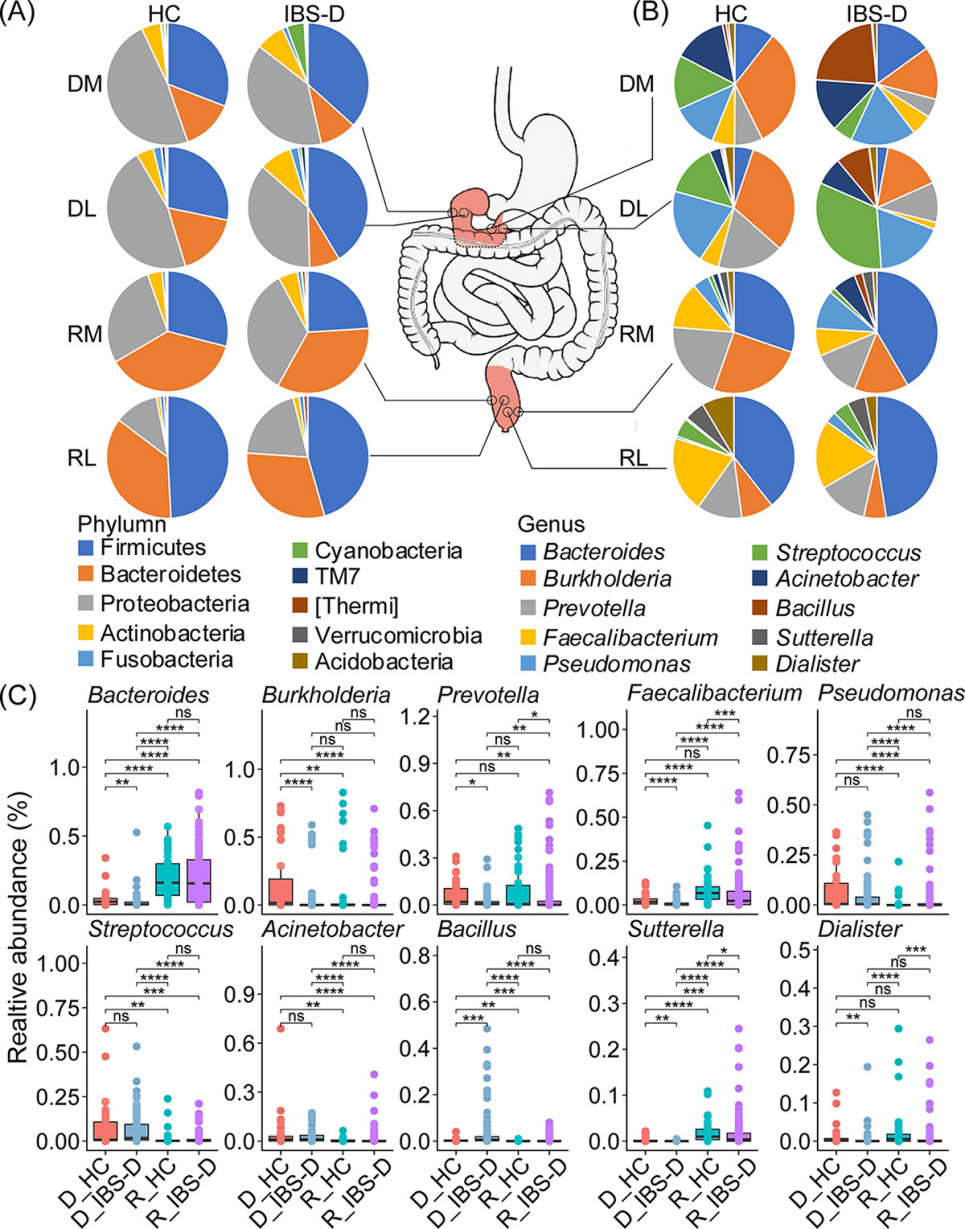
Comparison of microbial composition and enrichment patterns between HC and IBS-D patients in multiple intestinal sites. Composition of the top 10 microbes at (A) phylum level and (B) genus level across intestinal sites and hosts. (C) Comparison of relative abundance of the top 10 genera between duodenum and rectum of HC and IBS-D patients. Kruskal-Wallis was used to detect the global difference, while Wilcoxon test was used to detect the variation across intestinal sites in HC and IBS-D by pairwise comparisons. *, *P* < 0.05; **, *P* < 0.01; ***, *P* < 0.001; ****, *P* < 0.0001; ns, not significant. HC, healthy controls; IBS-D, diarrhea-predominant irritable bowel syndrome. DL, duodenal lumen; DM, duodenal mucosa; RL, rectal lumen; RM, rectal mucosa. D_HC, duodenal samples collected from HC; D_IBS-D, duodenal samples collected from IBS-D patients; R_HC, rectal samples collected from HC; R_IBS-D, rectal samples collected from IBS-D patients.

To uncover the site-specific microbial community patterns, the microbial composition of IBS-D and HC samples collected from DM, DL, RM, and RL was illustrated at phylum level (Fig. S5) and genus level (Fig. S6). Microbial communities were dominated by the bacterial phyla Firmicutes (average relative abundance 39.64%), Proteobacteria (average relative abundance 24.13%), and Bacteroidetes (average relative abundance 21.95%), while their relative abundances in each site were different (Fig. 3A), especially between duodenum and rectum (Fig. S5 and Fig. S7).

At genus level, microbial compositions of duodenum were different from those of rectum in both HC and IBS-D samples (Fig. 3B). For duodenum, we detected Pseudomonas, Streptococcus, and Acinetobacter were significantly enriched in this site, while *Burkholderia* and *Bacillus* were significantly enriched in HC samples and IBS-D samples, respectively (*P* < 0.05, Fig. 3C and Table S2). For rectum, *Faecalibacterium* was significantly enriched in HC samples (*P* < 0.1, Fig. 3C). In IBS-D patients, *Bacteroides*, a genus belonging to phyla Bacteroidetes, was found to be significantly abundant in rectum (Fig. 3C and Table S2), while it was deficient in DL.

### IBS-D patients have lower connectivity of microbial co-abundance network.

**FIG 4:**
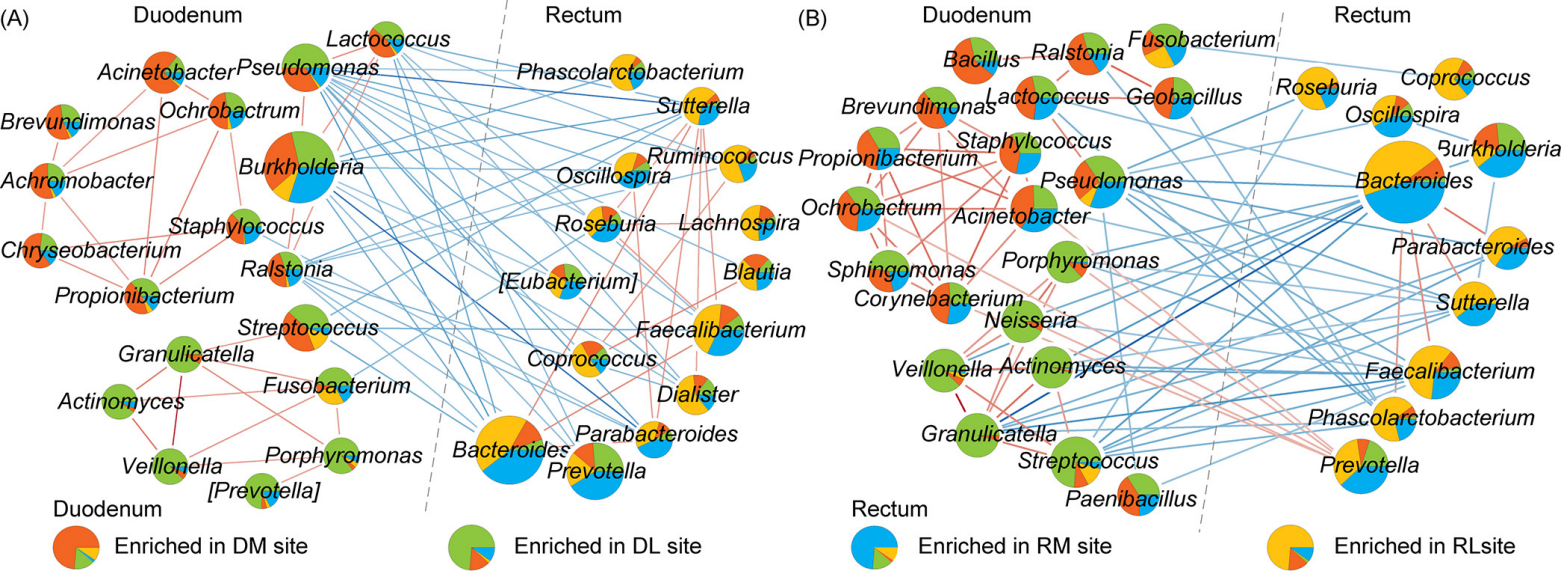
Genus-level co-abundance network of duodenum and rectum for HC and IBS-D microbial communities. Co-occurrence network of duodenum and rectum for (A) HC and (B) IBS-D microbial communities. Co-occurrence relationships between genera with Spearman correlations coefficient > 0.5 or <−0.2 and statistical significance (*P* < 0.05) were considered as strong correlations between genera and depicted in the Cytoscape. Nodes and edges represented microbial genera and their correlations, respectively. The size of nodes represents the relative abundance of genera. Red and blue edges represent positive and negative correlations, respectively. Each network was divided into two clusters: (left) cluster of duodenum-enriched genera and (right) cluster of rectum-enriched genera, according to the relative abundance of genera in duodenum and rectum. When DM and DL (or RM and RL) owned more proportion of a genus, this genus would be classified into the duodenal cluster (or rectal cluster).

Decreased microbial co-abundance network connectivity was observed in IBS-D patients compared with HC at genus level (Fig. S8). Most correlations were positive 51.28% for HC microbial community (Fig. 4A) and 53.01% for IBS-D microbial community (Fig. 4B) within the cluster of duodenum-enriched genera and the cluster of rectum-enriched genera. However, several correlations were negative between the clusters dominated by duodenum-enriched genera and rectum-enriched genera. From these networks, we have also observed several genera whose relative abundances and connectivities were different among the four intestinal sites in IBS-D patients. For example, in IBS-D patients, *Bacteroides*, a genus enriched in rectum, has strong positive correlations (Spearman correlation coefficient > 0.4) with *Parabacteroides* and *Phascolarctobacterium* (Fig. 4B), which were potentially associated with intestinal inflammation (23–25). However, in HC samples, such profound correlations among these two genera and *Bacteroides* cannot be detected (Fig. 4A). Moreover, although most species of *Bacteroides* are commensals, several species of *Bacteroides* were considered to be pathobionts that can become pathogenic under specific environmental factors, such as antibiotic resistance usage (26, 27). In this network, *Prevotella* was negatively correlated with several genera in HC network, while it was positively correlated with other genera in the IBS-D patients (Fig. 4). Its colonization in the intestine could reduce the production of interleukin 18 (IL-18) (28), and thus probably exacerbate intestinal inflammation, and potential systemic autoimmunity (29).

### IBS-D patients could be accurately predicted based on site-specific biomarkers.

**FIG 5:**
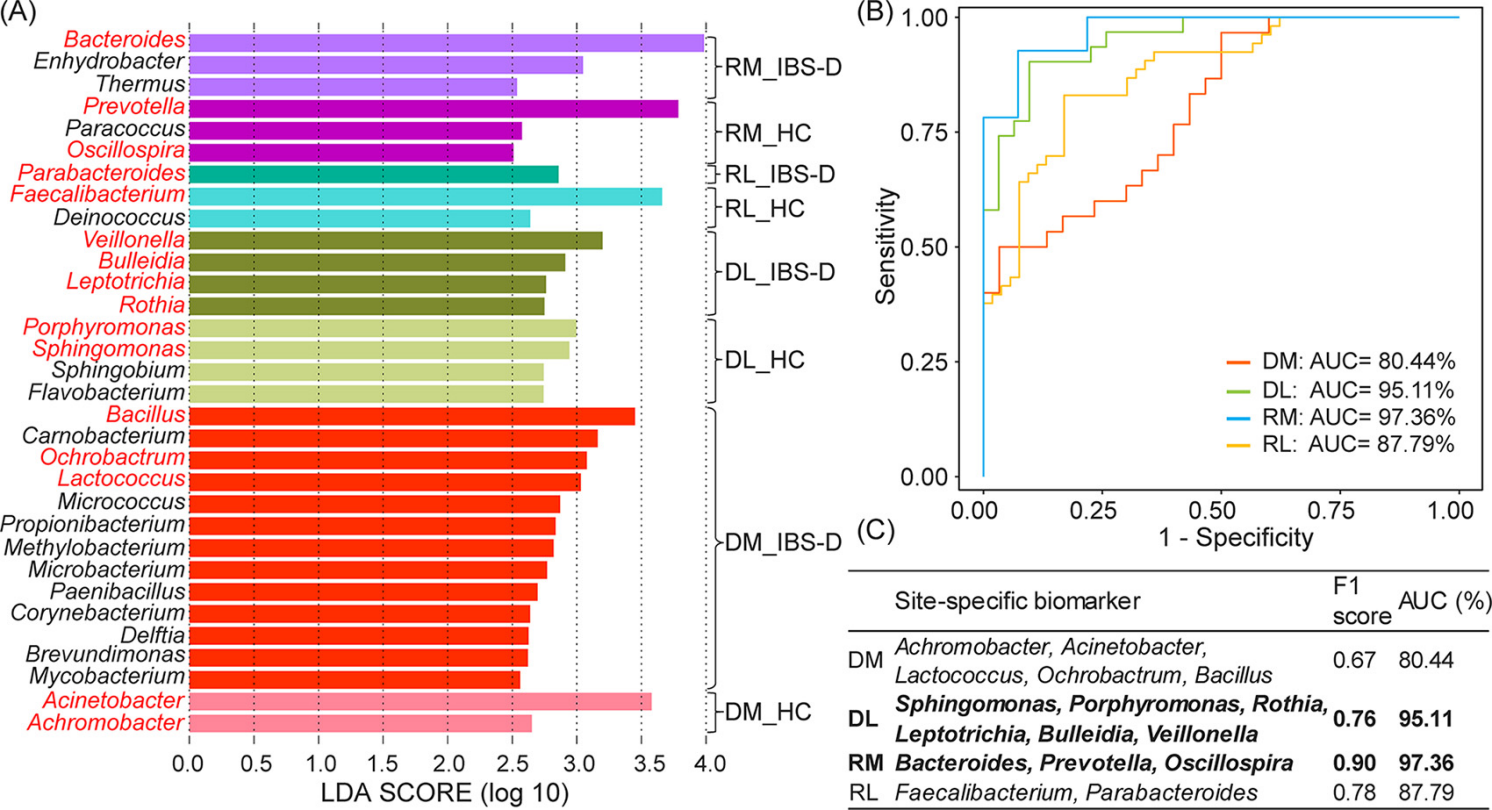
Several combinations of site-specific biomarkers could be used for the prediction of host health status with high fidelity. (A) Site-specific biomarkers detected by LEfSe. (B) The performances of site-specific biomarkers using AUC measures. (C) List of site-specific biomarkers and comparison of their prediction accuracies using F1 score and AUC measures. HC, healthy controls; IBS-D, diarrhea-predominant irritable bowel syndrome. DL, duodenal lumen; DM, duodenal mucosa; RL, rectal lumen; RM, rectal mucosa. DL_HC, duodenal luminal samples collected from HC; DL_IBS-D, duodenal luminal samples collected from IBS-D; DM_HC, duodenal mucosal samples collected from HC; DM_IBS-D, duodenal mucosal samples collected from IBS-D; RL_HC, rectal luminal samples collected from HC; RL_IBS-D, rectal luminal samples collected from IBS-D; RM_HC, rectal mucosal samples collected from HC; RM_IBS-D, rectal mucosal samples collected from IBS-D. AUC, area under receiver operating curve.

The site-specific biomarkers, especially for biomarkers from RM and DL, could be used for IBS-D prediction with high fidelity. We identified 32 site-specific biomarkers by LEfSe method with LDA > 2.5 (list in Fig. 5A). Among them, 15 biomarkers were identified for DM (HC: two, IBS-D patients: 13), eight biomarkers for DL (HC: four, IBS-D patients: four), six biomarkers for RM (HC: three, IBS-D patients: three), and three biomarkers for RL (HC: two, IBS-D patients: one).

To find out which site-specific biomarkers could best characterize the microbial community of IBS-D patients, a series of Random Forest (RF) models were built based on these site-specific biomarkers. For DM, the combination of *Achromobacterr*, Acinetobacter, *Lactococcus*, *Ochrobactrum*, and *Bacillus* could differentiate the HC and IBS-D samples with 80.44% area under receiver operating characteristic curve (AUC) (F1 = 0.67, Fig. 5B and C, red curve). For DL, the combination of *Porphyromonas*, *Sphingomonas*, *Veillonella*, *Bulleidia*, *Leptotrichia*, and *Rothia* showed outstanding performance with 95.11% AUC (F1 = 0.76, Fig. 5B and C, green curve). The combination of RM site-specific biomarkers including *Bacteroides*, *Prevotella* and *Oscillospira*, could be used to precisely distinguish whether the participant is an IBS-D patient (F1 = 0.90 and AUC = 97.36%, blue curve in Fig. 5B and C, and Fig. S9). For RL, according to the biomarkers’ performance, we can use *Parabacteroides* and *Faecalibacterium* to accurately differentiate IBS-D patients (F1 = 0.78 and AUC = 87.79%, Fig. 5B and C, yellow curve). Collectively, RM site-specific biomarkers showed the best performance compared with other intestinal sites, followed by DL, indicating the strong potential of RM biomarkers to serve as biomarkers for next-generation IBS-D diagnosis.

Again, RM’s site-specific IBS-D biomarker *Bacteroides* was included in the RF model, which also showed outstanding performance in differentiating IBS-D patients from HC (F1 = 0.84 and AUC = 91.01%, Fig. S9), confirming its key roles in IBS-D (30). Furthermore, among all sub-combinations of *Bacteroides*, *Prevotella*, and *Oscillospira*, the combination of *Bacteroides* and *Prevotella* could best differentiate the IBS-D patients from HC (F1 = 0.83 and AUC = 99.44%, Fig. S9), followed by the combination of *Bacteroides* and *Oscillospira* (F1 = 0.86 and AUC = 93.80%). These results suggested the distinct advantage of using RM biomarkers *Bacteroides*, *Prevotella* and *Oscillospira* to identify the IBS-D patients.

### The site-specific biomarkers have profound associations with clinical symptoms in IBS-D patients.

**FIG 6:**
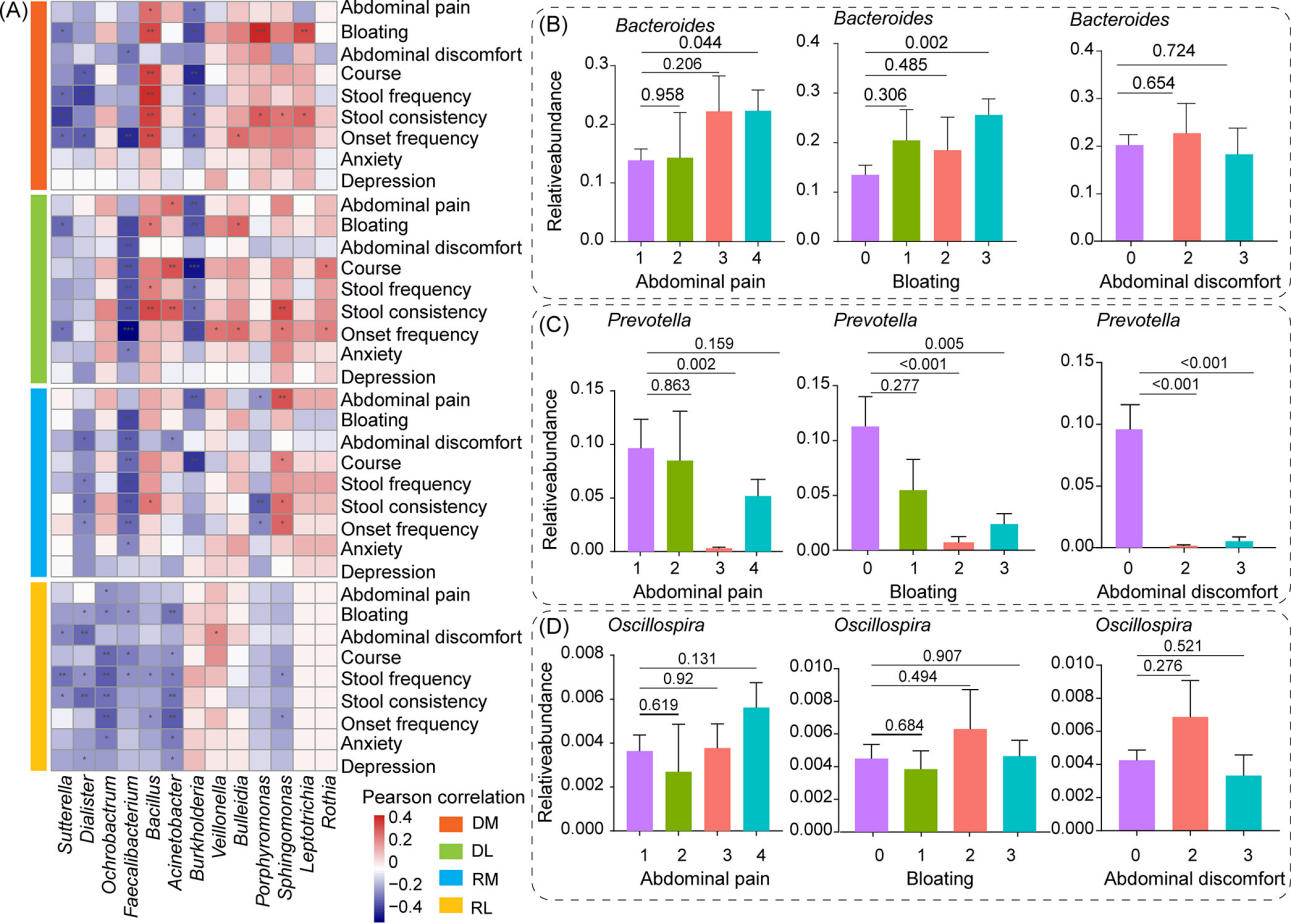
Microbial genera have profound associations with clinical symptoms in IBS-D patients. (A) Pearson correlations between clinical symptoms and microbial genera of DM, DL, RM and RL in IBS-D patients. Association between the severity of abdominal pain, bloating, abdominal discomfort and the relative abundance of (B) *Bacteroides*, (C) *Prevotella*, and (D) *Oscillospira* in RM. Only the adjusted *P value* < 0.05 was marked as “*” in Fig. 6A. Analysis of variance with least-significant-difference (ANOVA-LSD) was used to detect the differences between different groups in Fig. 6B. HC, healthy controls; IBS-D, diarrhea-predominant irritable bowel syndrome; DM, duodenal mucosa; DL, duodenal lumen; RL, rectal lumen; RM, rectal mucosa.

Strong associations were observed among bacterial genera in four intestinal sites and clinical symptoms of IBS-D patients. Genera *Bacillus* and *Sphingomonas* in DM, DL, and RM were identified with positive correlations with abdominal symptoms (list in Fig. 6A) of IBS-D patients, such as the severity of abdominal pain, bloating, stool consistency (*P* < 0.05). On the contrary, *Faecalibacterium*, *Dialister*, and *Sutterella* in all intestinal sites were negatively correlated with clinical symptoms of IBS-D patients (*P* < 0.05), such as the severity of abdominal pain, bloating, and stool consistency (Fig. 6A).

Considering the high prediction power of RM biomarkers including *Bacteroides*, *Prevotella*, and *Oscillospira* in identifying IBS-D patients, we also explored their association with clinical symptoms: the severity of abdominal pain, bloating, and abdominal discomfort. Notably, the increasing severity of abdominal pain and bloating was accompanied by a rising abundance of *Bacteroides* (Fig. 6B). The abundance of *Prevotella* was decreased when the severity of abdominal pain, bloating, and abdominal discomfort were aggravated (Fig. 6C). However, no significant association was detected between abdominal symptoms and *Oscillospira* in RM (Fig. 6D). Furthermore, based on RF model built by RM site-specific biomarkers, the misclassified IBS-D patients showed weaker clinical symptoms compared with patients that were classified correctly (Fig. S10). These results further confirmed the important role of RM site-specific biomarkers *Bacteroides*, *Prevotella*, and *Oscillospira* in IBS-D.

## DISCUSSION

This study collected the largest number of samples from multiple intestinal sites of IBS-D patients and HC in China, aiming to generate a holistic understanding of mucosa and lumen-associated microbial profiles in duodenum and rectum of IBS-D patients and HC, as well as the potential role of RM microbes in IBS-D. The novel discoveries of this study could be understood in three contexts.

Firstly, microbial communities from different intestinal sites have different microbial compositions, largely due to the colonization of bacteria in different intestinal sites. This result again confirmed the fecal microbial community could not fully represent the intestinal microbial community (13). IBS-D patients and HC also have significantly different representative species even at the same intestinal sites. *Bacillus*, *Burkholderia*, *Prevotella*, and *Faecalibacterium* were the representative genera in duodenum of IBS-D, duodenum of HC, rectum of IBS-D, and rectum of HC, respectively. The reduction of *Burkholderia* in duodenum of IBS-D could affect the colonization of the probiotic strain (31), and thus facilitate intestinal inflammation (32). *Faecalibacterium* was reduced in IBS-D patients at RM site compared with HC (8, 10). It was reported that *Faecalibacterium* could produce butyrate (33) and anti-inflammatory protein (34), which could improve intestinal immunity and epithelial barrier (35). Hence, its reduction might contribute to intestinal inflammation, and thus promote the onset of IBS-D (36, 37). On the contrary, the relative abundance of *Bacillus* was increased in duodenum in IBS-D patients compared with HC. *Bacillus* could secret acetylcholine, which may potentially lead to abdominal pain and diarrhea in IBS-D patients (38, 39).

Secondly, we emphasize the importance of RM microbial community in IBS-D. RF modeling based on RM site-specific biomarkers *Bacteroides*, *Prevotella*, and *Oscillospira* could yield high prediction accuracy (F1 = 0.90 and AUC = 97.36%) for better diagnosis of IBS-D patients. However, fecal (RL) microbial biomarkers *Parabacteroides* and *Faecalibacterium* (F1 = 0.78 and AUC = 87.79%) could not perform as well as RM. Hence, rather than fecal samples (12, 13, 40). RM samples were able to reflect more predominant characteristics of intestinal microbial community of IBS-D patients. This phenomenon was consistent with a published study in analyzing intestinal microbial community of naive pediatric Crohn’s disease using ileal, rectal, and fecal samples (41). Moreover, the biomarkers detected by LEfSe in other intestinal sites were also important. For example, the biomarkers of DL (*Porphyromonas*, *Sphingomonas*, *Veillonella*, *Bulleidia*, *Leptotrichia*, and *Rothia*) showed an outstanding performance in identifying IBS-D patients (AUC = 95.11%, F1 = 0.76), and its predictive ability was second to RM. Notably, *Porphyromonas*, *Veillonella*, and *Leptotrichia* were reported to promote intestinal dysbiosis by influencing microbial communities (42). Additionally, the misclassified IBS-D patients by RF model using RM site-specific biomarkers, were also confirmed with weaker clinical symptoms compared with these correctly classified patients (Fig. S10), while some of these misclassified IBS-D patients could be correctly identified by the RF model using DL site-specific biomarkers (Table S3). All of these results emphasized the importance of RM microbial community in IBS-D. In summary, this is the first study to use the RM site-specific biomarkers *Bacteroides*, *Prevotella*, and *Oscillospira* to build a prediction model for the better diagnosis of IBS-D patients. These results also highlight the importance for exploring the potential link between IBS-D and RM microbial community.

Thirdly, RM site-specific biomarker *Bacteroides* was a genus that could serve well for fast and easy IBS-D diagnosis. The co-occurrence network has also shown its alteration in IBS-D patients compared with HC. In IBS-D patients, *Bacteroides* was positively correlated with *Parabacteroides* and *Phascolarctobacterium*, which were reported as intestinal inflammation contributors (23–25). This alteration might also indicate the potential role of *Bacteroides* in the pathophysiology of IBS-D. Using *Bacteroides* alone or together with *Prevotella* and *Oscillospira* from RM could result in high identification accuracy for IBS-D patients, among all RF models generated in this study, confirming its key role in IBS-D (30). As previously described (11, 43, 44), *Bacteroides* was a genus enriched in IBS-D patients, which was also found strongly associated with the severity of abdominal pain and bloating. Though this genus contains numerous commensal species that could secret immunomodulatory factors to regulate intestinal inflammation, such as Bacteroides vulgatus (45) and Bacteroides ovatus (46), several species of this genus could produce enterotoxins, such as Bacteroides fragilis (47) and Bacteroides melaninogenicus (48). Among them, Bacteroides fragilis could bind to IgA for colonization in intestinal mucosa, which might destroy the intestinal epithelial barrier and alter gut motility, leading to abdominal pain or bloating (49–51). This species was also resistant to antibiotics (52) and associated with diarrhea (50, 51). Taken together, monitoring *Bacteroides* from RM, though it might incur invasive sampling, would serve well in the next-generation diagnosis of IBS-D patients.

This study also has limitations. Firstly, the relatively small sample size prevented the extrapolation of our results to the general IBS-D population. However, considering the difficulty of endoscopy and biopsy for participants who offered their samples collected from multiple intestinal sites, this study has tried our best to collect the largest number of samples from multiple intestinal sites of IBS-D patients and HC in China. Secondly, our study revealed the site-specific microbial patterns and found the discriminative microbes for IBS-D diagnosis, although diet (such as high-fat diet) and lifestyle (such as smoking) play crucial roles in shaping intestinal microbial communities (53–55). Based on our analysis, further studies could explore the contribution of these factors to intestinal microbiota and the pathogenesis of IBS-D. Thirdly, due to the difference of intestinal microbial communities between Chinese and western people, most of the publicly available IBS-D data were from western individuals, the external validation test using the public data set was largely limited. With the increasing number of samples from multiple cohorts becoming available, universal patterns of intestinal microbial communities for IBS-D patients might be better understood.

### Conclusion.

To conclude, our study collected the largest number of samples from multiple intestinal sites of IBS-D patients and HC in China. We found that IBS-D microbial community has specific patterns at each intestinal site differing from that of HC. Fecal microbial communities (that is RL samples) could not fully represent the intestinal microbial communities. Additionally, IBS-D microbial co-abundant network has lower interactions compared with HC. Moreover, using genera *Bacteroides* alone or together with *Prevotella* and *Oscillospira* of RM was found to have high prediction power to differentiate IBS-D from HC. Furthermore, *Bacteroides* and *Prevotella* in RM were closely related to the severity of abdominal pain, bloating, and abdominal discomfort in IBS-D patients.

Considering that most researches about the intestinal microbial community in IBS focused on fecal microbial community, this study is of particular importance in helping researchers understand the great potential of the microbial community in RM for better diagnosis of IBS-D. To better understand this potential, further researches are necessary to figure out the importance of the enrichment of *Bacteroides*, and decrease of *Prevotella* and *Oscillospira* in RM for IBS-D. All of these ongoing and future efforts could contribute to the realization of the next-generation IBS-D diagnosis.

## MATERIALS AND METHODS

### Ethics approval.

This study was approved by the Institutional Ethical Review Committee of Huazhong University of Science and Technology (2014-196) and Chinese Clinical Trials Registry (registration number: ChiCTR-OPC-15007624).

### Study design and sample description.

Healthy individuals and IBS-D patients aged 35.89 on average (standard deviation:10.49), who expressed interest were invited to participate in this study, at the Division of Gastroenterology, Union Hospital, Tongji Medical College, Huazhong University of Science and Technology. For IBS-D patients, they were diagnosed according to Rome III criteria (17). Participants with the following situations were excluded from this study: (i) use antibiotics, prebiotics, probiotics, proton-pump inhibitors, colon-cleansing drugs, or any medications for IBS-D within a month before the start of this study; and (ii) acute gastroenteritis, coeliac disease, inflammatory bowel diseases, cancer, severe systemic diseases, abdominal surgery history, and/or psychiatric diseases. Healthy participants without personal history of immune-related diseases or gastrointestinal complaints were recruited as a control group. In total, this study recruited 74 IBS-D patients and 20 healthy participants.

Each participant signed informed consent and completed a questionnaire that included their clinical symptoms (Table S1). These clinical symptoms mainly included age, body mass index (BMI), stool consistency based on Bristol Stool Form (BSF) (56), frequency of defecation per day, onset frequency, course of IBS-D, the severity of abdominal pain, abdominal discomfort and bloating, and psychological state according to Hospital Anxiety and Depression (HAD) scale (57). The severity of abdominal pain, abdominal discomfort, and bloating were classified into not at all, mild, moderate and severe, which were represented by numbers 0, 1, 2, and 3, respectively. Onset frequency was divided into once or twice a month, three times a month, once a week, more than once a week and every day, which were represented by numbers 1, 2, 3, 4, and 5, respectively.

We investigated microbial profiles at multiple intestinal sites of IBS-D patients and HC, including DM, DL, RM, and RL samples. DM samples were collected by endoscopy from duodenum about 10 cm below the major duodenal papilla using biopsy forceps under the endoscope. DL samples were collected from the fluid in the same position with DM endoscopically using a sterile catheter. RM samples were collected from the rectum approximately 10 cm above the anus using biopsy forceps under the endoscope. RL samples (in this study, we collected fecal samples from rectum, and considered the fecal samples as RL samples) were obtained from participants after excretion as soon as possible. Then, these samples were carefully packed into sterile Eppendorf tubes and placed on ice. After that, these samples were immediately transported to the laboratory and stored at −80°C within 30 min. For mucosal biopsies, the HE staining has shown there was no inflammatory infiltration in all histological sections under the microscope (Fig. S11). We totally collected 283 samples from these four representative intestinal sites (Table S4). Among them, IBS-D samples contained 37 DM samples, 43 DL samples, 53 RM samples, and 74 RL samples. HC samples included 20 DM samples, 19 DL samples, 20 RM samples, and 17 RL samples (Table S4). The vast majority of participants contributed samples from at least two sites (77.66%), while 22.34%, 8.51%, 14.89%, and 54.26% of patients contributed one, two, three, and four sites of intestinal samples, respectively. All samples were preserved at −80°C until performing 16S rRNA gene sequencing.

### DNA extraction and 16S rRNA gene sequencing.

DNA of all samples was extracted using a Fast DNA SPIN Kit (Tiangen Biotech). Then, the universal primers (forward: 5′-AGAGTTTGATCCTGGCTCAG-3′, reverse: 5′-TTACCGCGGCTGCTGGCAC-3′) and fusion primers (forward: 5′-454adapter-mid-AGAGTTTGATCCTGGCTCAG-3′, reverse: 5′-454adapter-TTACCGCGGCTGCTGGCAC-3′) were used to amplify the 16S rRNA V1-V3 regions. After that, all products were sent for 16S rRNA gene sequencing on a 454 Life Sciences Genome Sequencer FLX platform with titanium (Roche Life Sciences). Finally, all of these 283 samples were successfully sequenced for subsequent analysis.

### Taxonomy annotation and microbial diversity analysis.

The low-quality reads which contained ambiguous base calls (N), or less than 300 bp were filtered using QIIME (version 1.9.1) (58). The primers were removed using the mothur (59) commands “chimera.uchime” and “remove.seqs” based on Silva database (Release 123) (60). These high-quality sequences were clustered into operational taxonomic units (OTUs) at 97% sequence similarity using QIIME (58) script pick_de_novo_otus.py. For OTUs, we filtered out the OTUs only detected in one sample with one read, and then these OTUs were assigned to the Greengenes database for taxonomy classification (61).

Before microbial diversity analysis, we subsampled all the samples to 187,000 reads using the QIIME script single_rarefaction.py (parameter: -d 187000), given the different sequences for each sample. Alpha (measured by Shannon index) and beta (measured by unweighted UniFrac and Bray Curtis distance) diversity metrics were used to analyze the microbial diversity using QIIME (58). Principal coordinates analysis (PCoA) based on Jaccard coefficient was used to determine the discrepancies between HC and IBS-D, or between different intestinal sites using R package “vegan.” Kruskal-Wallis was used to detect the global difference, while the Wilcoxon test was applied to detect the variation between HC and IBS-D microbial communities or the variation across intestinal sites by paired comparisons. The false discovery rate (FDR) adjusted *P value* was calculated using the Benjamini and Hochberg (BH) method. Procrustes analysis was utilized to assess the similarity of the two compared microbial communities based on R procrustes() function, and the results were tested by the Monte Carlo method with 999 permutations.

### Co-abundant network analysis.

For co-abundance network analysis, we first selected the genera with relative abundance no less than 0.5% and coverage of at least 10% samples. Secondly, these genera were utilized for co-occurrence network analysis using Spearman correlation as correlation measurement. Thirdly, the co-occurrence relationships among these microbes with *P value* < 0.05 were considered as significant correlations (Spearman correlation coefficient: larger than 0.5 or smaller than −0.2), and depicted in the network diagram with genera as nodes and their correlations as edges (visualized by Cytoscape) (62). The FDR adjusted *P value* was calculated by BH method. Each network was divided into two clusters according to the abundance of the genera: When a genus was detected with a higher proportion of relative abundance in DM and DL, this genus would be classified into the duodenal cluster, or this genus was assigned to the rectal cluster.

### Prediction model for distinguishing IBS-D patients and healthy participants.

To detect the species that could be used for better diagnosis of IBS-D patients, LEfSe (63) was utilized to identify the site-specific biomarker with LDA > 2.5. Then, a RF model (64) was developed to distinguish the IBS-D samples from HC based on these site-specific biomarkers. For the prediction model, important parameters ntree (number of decision trees in RF model) and mtry (variable sampling value for each iteration) were trained and estimated by the out-of-bag (OOB) values using R package “randomForest” (64). This process was iterated 15,000 times to construct the accurate model without overfitting. We also used the AUC and F1 score to evaluate the performance of the prediction model.

### Association of intestinal bacteria and clinical symptoms in IBS-D patients.

Pearson correlation analysis was conducted to detect the association of intestinal bacteria and clinical symptoms (Table S1). The FDR adjusted *P value* was calculated using BH method. Only correlations identified with adjusted *P* < 0.05 were considered as significant correlations and visualized in the heatmap.

### Data availability.

All the raw sequencing data in this study are available in the Genome Sequence Archive (GSA) database (GSA accession number: PRJCA004584), which can be accessed at https://bigd.big.ac.cn/bioproject/browse/PRJCA004584.
