# Supplementary material for: Understanding of the Site-Specific Microbial Patterns towards Accurate Identification for Patients with Diarrhea-Predominant Irritable Bowel Syndrome
Source: Microbiol Spectr. 2021 Dec 22;9(3):e01255-21. doi: 10.1128/Spectrum.01255-21 (PMC8694097; doi:10.1128/Spectrum.01255-21)
Supplement: SUPPLEMENTAL FILE 1 — Supplemental material. Download SPECTRUM01255-21_Supp_1_seq8.pdf, PDF file, 1.9 MB [file spectrum01255-21_supp_1_seq8.pdf]

Supplementary Materials

Understanding of the site-specific microbial patterns towards accurate identification for patients with diarrhea-predominant irritable bowel syndrome

Running title: RM site-specific microbes are critical in IBS-D

Xue Zhu[1,*], Gaichao Hong[2,*], Ying Li[2], Pengshuo Yang[1], Mingyue Cheng[1], Lei Zhang[2], Yuxue Li[1], Lei Ji[1], Gangping Li[2], Chaoyun Chen[1], Chaofang Zhong[1], Yu Jin[2], Min Yang[2], Hanhua Xiong[2], Wei Qian[2], Zhen Ding[2,#], Kang Ning[1,#], Xiaohua Hou[2,#]

[1]Key Laboratory of Molecular Biophysics of the Ministry of Education, Hubei Key Laboratory of Bioinformatics and Molecular-imaging, Center of AI Biology, Department of Bioinformatics and Systems Biology, College of Life Science and Technology, Huazhong University of Science and Technology, Wuhan 430074, Hubei, China

[2]Division of Gastroenterology, Union Hospital, Tongji Medical College, Huazhong University of Science and Technology, Wuhan 430022, China

[*]Xue Zhu and Gaichao Hong contributed equally to this study

[#]**Correspondence to** Dr Xiaohua Hou, E-mail: houxh@hust.edu.cn; Dr Kang Ning, E-mail: ningkang@hust.edu.cn; Dr Zhen Ding, E-mail: docd720@126.com

Supplementary Figures and Tables

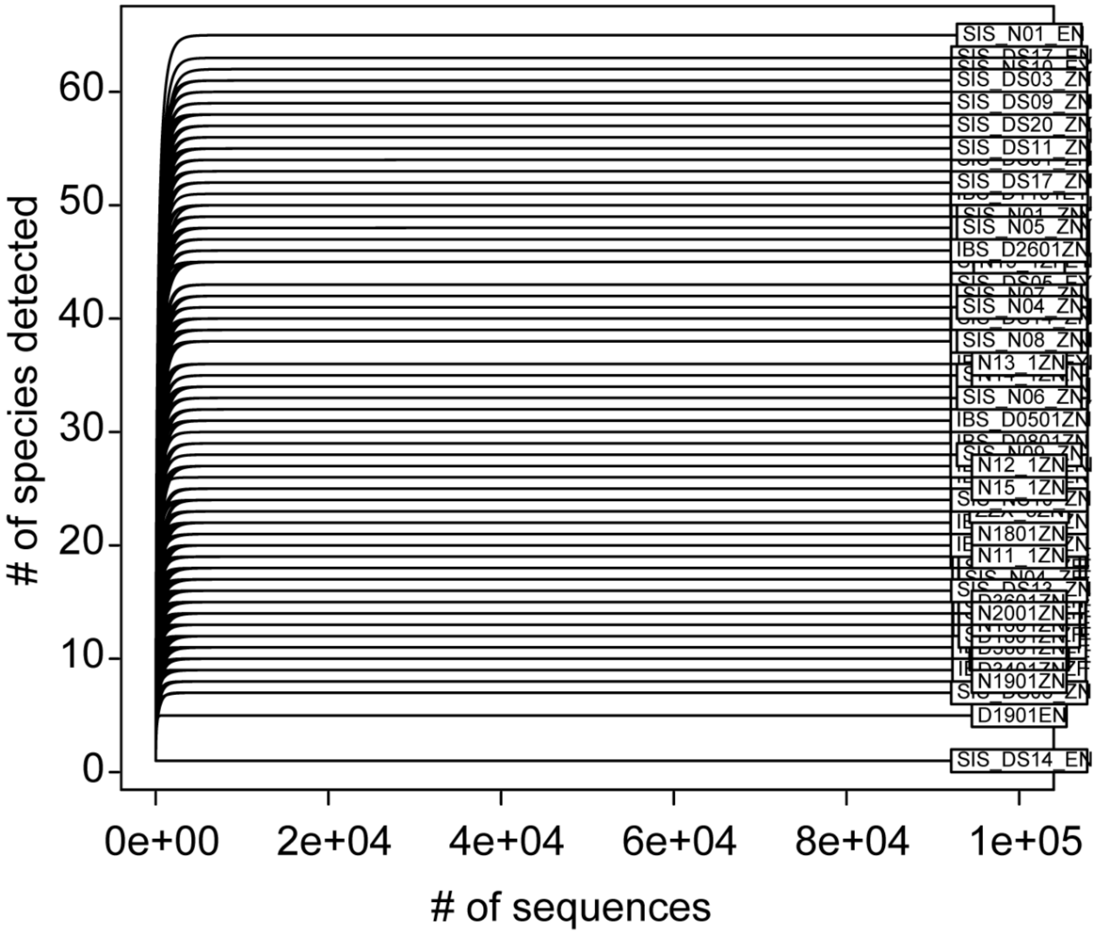

Supplementary Figure 1. Rarefaction curves of sequencing depth for samples collected from IBS-D patients and healthy participants. The number of species detected in each sample (y-axis) was increased with the increasing number of sequences per sample (x-axis). All curves showed saturation at around 10,000 sequences per sample, suggesting the sequencing depth is enough to capture all species.

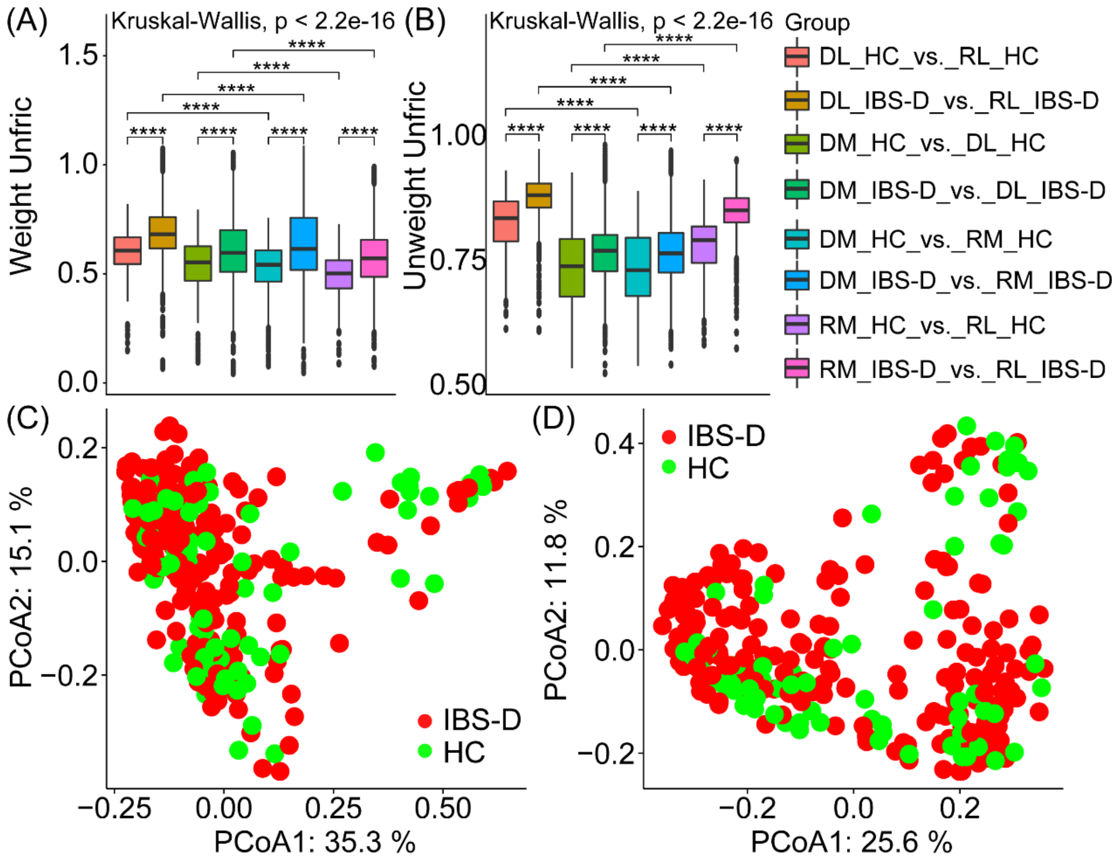

Supplementary Figure 2. Comparison of beta diversity of microbial communities across intestinal sites and hosts based on (A) & (C) weight and (B) & (D) unweighted UniFrac distance. Kruskal-Wallis was used to detect the global difference, while the Wilcoxon test was used to detect variation across intestinal sites in HC and IBS-D patients based on the microbial composition at genus level. DL_HC: duodenal luminal samples collected from HC; DM_HC: duodenal mucosal samples collected from HC; RL_HC: rectal luminal samples collected from HC; RM_HC: rectal mucosal samples collected from HC; DL_IBS-D: duodenal luminal samples collected from IBS-D patients; DM_IBS-D: duodenal mucosal samples collected from IBS-D patients; RL_IBS-D: rectal luminal samples collected from IBS-D patients; RM_IBS-D: rectal mucosal samples collected from IBS-D patients. *: p < 0.1; **: p<0.05; ***: p < 0.01. ****: p < 0.001.

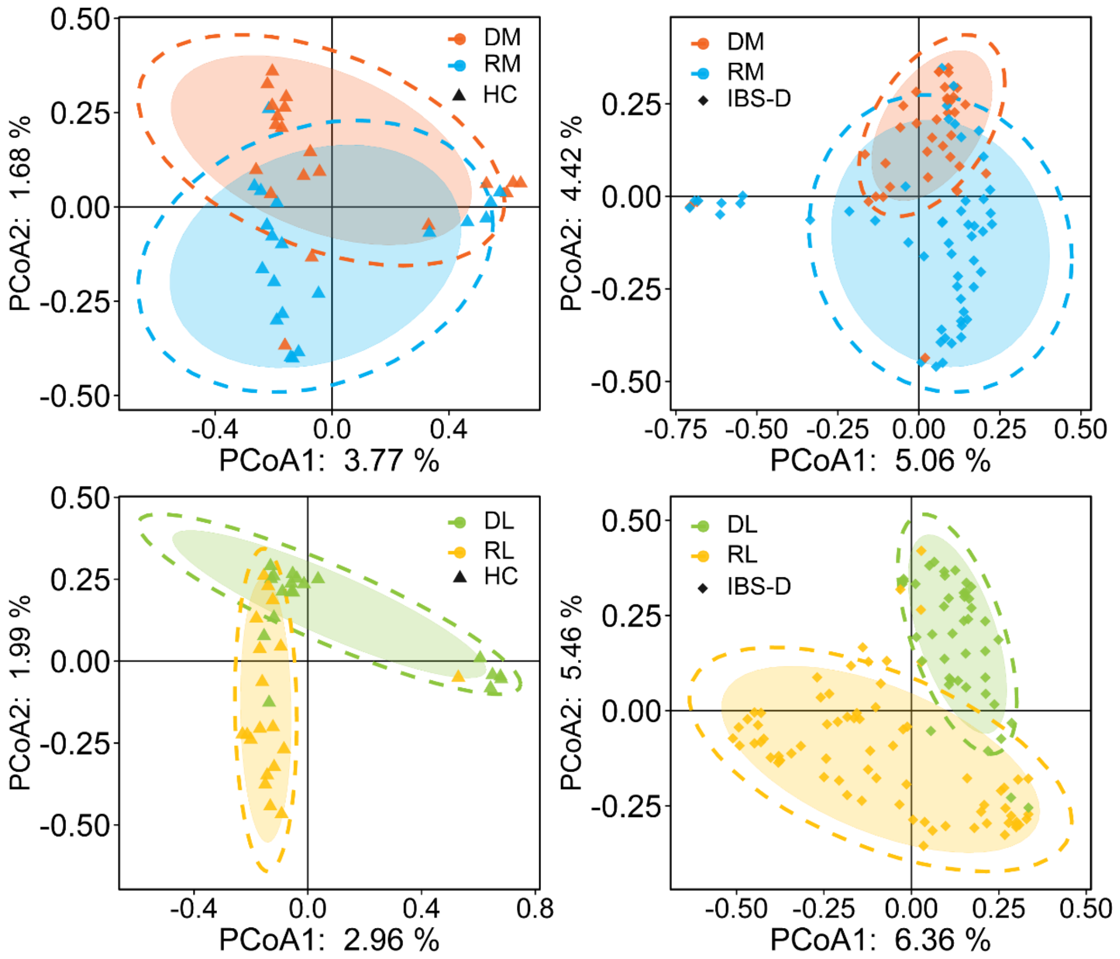

Supplementary Figure 3. Comparison of microbial communities between different intestinal sites. Comparison of microbial communities between DM and RM in all (A) HC and (B) IBS-D samples based on PCoA analysis with Jaccard coefficient as the distance measurement. Comparison of microbial communities between DL and RL in all (C) HC and (D) IBS-D samples. HC: healthy controls; IBS-D: diarrhea-predominant irritable bowel syndrome. DL: duodenal lumen; DM: duodenal mucosa; RL: rectal lumen; RM: rectal mucosa. M_HC: mucosal samples from HC; M_IBS-D: mucosal samples from IBS-D; L_HC: luminal samples from HC; L_IBS-D: luminal samples from IBS-D. These comparisons were based on microbial composition at genus level.

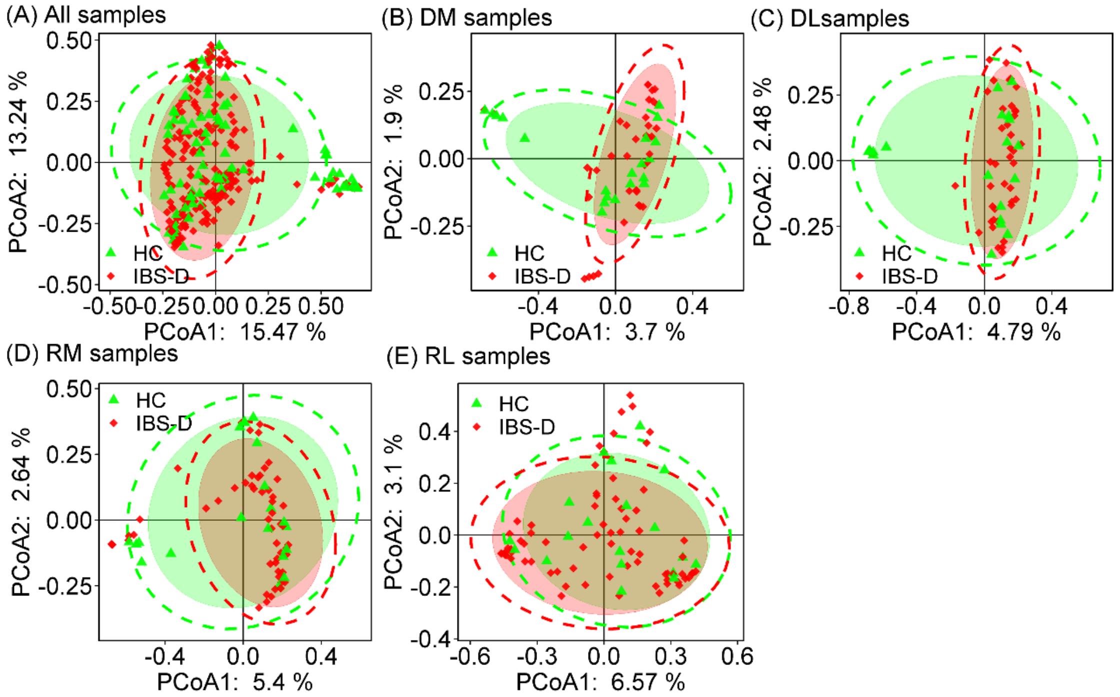

Supplementary Figure 4. Comparison of microbial communities across intestinal sites between healthy individuals and IBS-D patients. Comparison of microbial communities between HC and IBS-D based on (A) all samples, (B) DM samples, (C) DL samples, (D) RL samples and (E) RM samples. HC: healthy controls; IBS-D: diarrhea-predominant irritable bowel syndrome. DL: duodenal lumen; DM: duodenal mucosa; RL: rectal lumen; RM: rectal mucosa. These comparisons were conducted based on microbial composition at genus level.

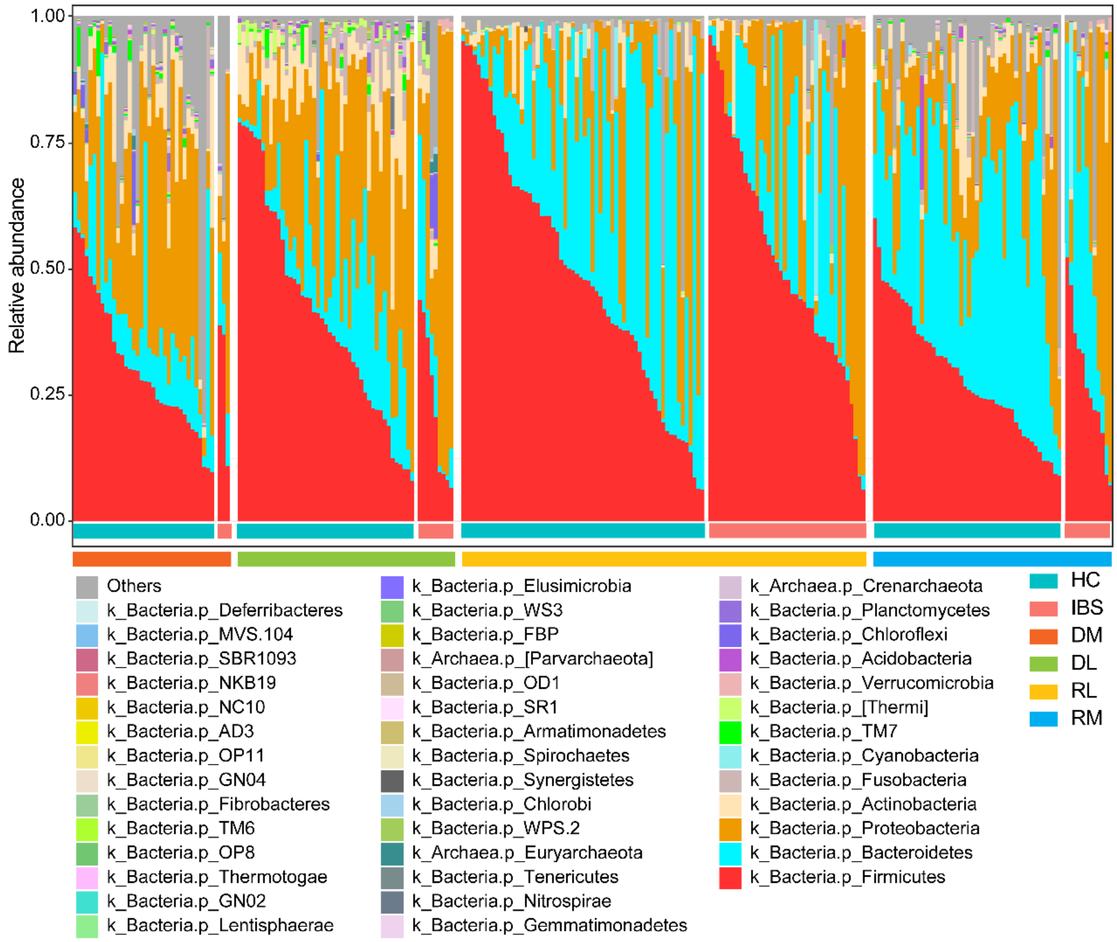

Supplementary Figure 5. Microbial composition across intestinal sites at phylum level. Only the top 30 phyla were depicted in this figure. Others mean the phylum except for the top 30 phyla. DM, DL, RL and RM are represented by orange, green, yellow and blue horizontal bar, respectively. HC: healthy controls; IBS-D: diarrhea-predominant irritable bowel syndrome; DM: duodenal mucosa; DL: duodenal lumen; RL: rectal lumen; RM: rectal mucosa.

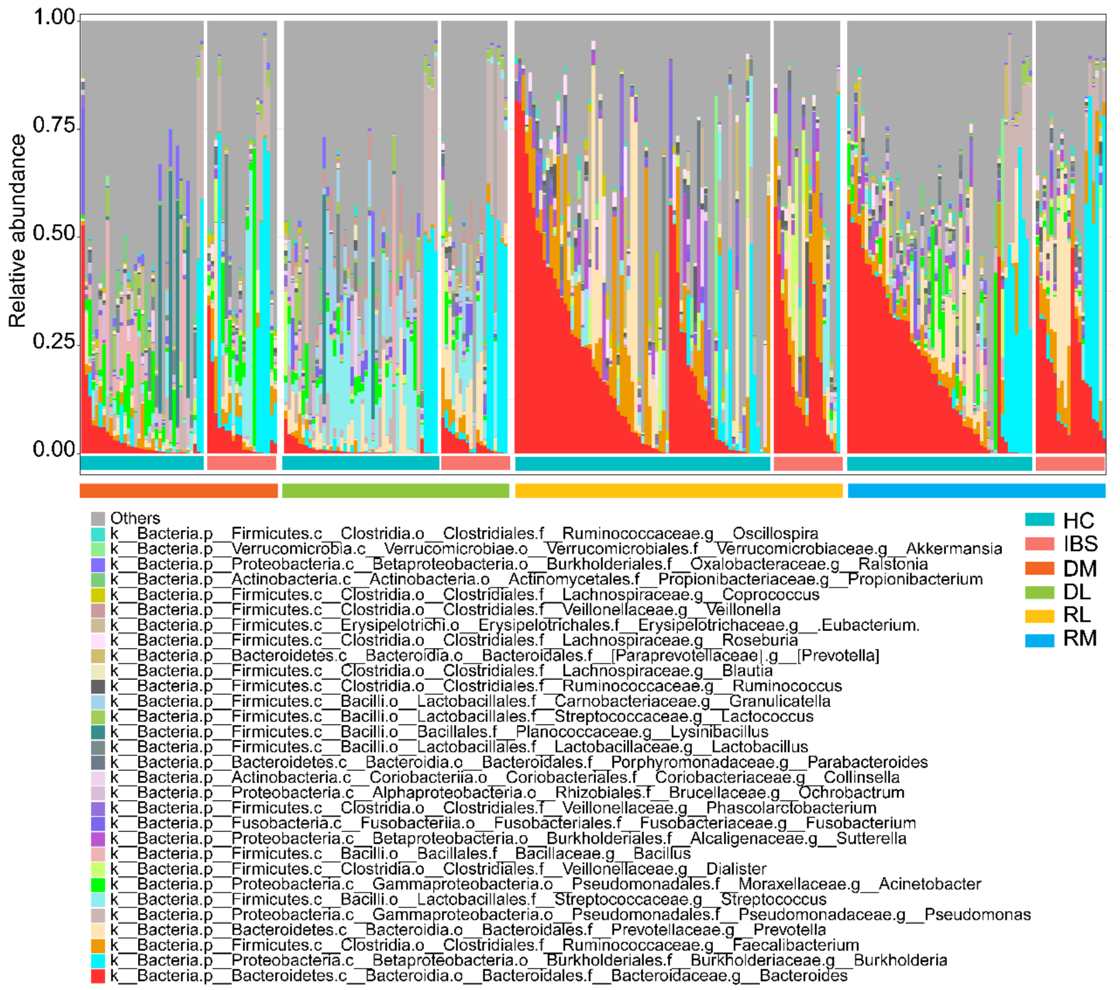

Supplementary Figure 6. Microbial composition across intestinal sites at genus level. Only the top 30 genera were depicted in this figure. Others mean the phylum except for the top 30 phyla. DM, DL, RL and RM are represented with orange, green, yellow and blue horizontal bars, respectively. HC: healthy controls; IBS-D: diarrhea-predominant irritable bowel syndrome; DM: duodenal mucosa; DL: duodenal lumen; RL: rectal lumen; RM: rectal mucosa.

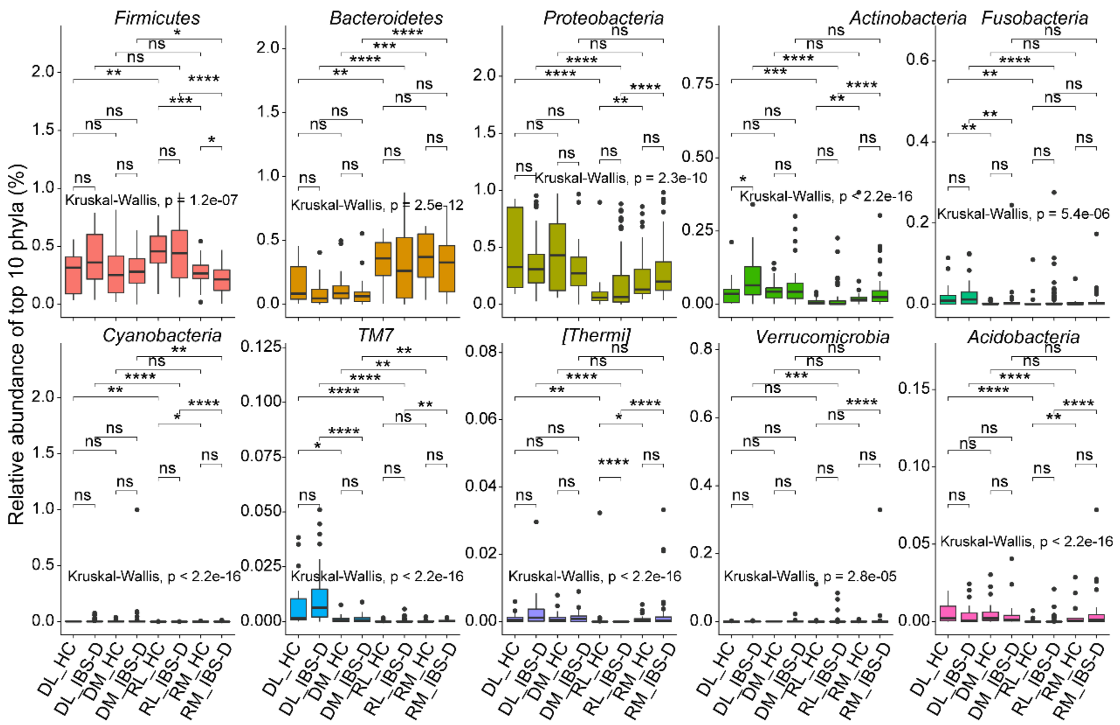

Supplementary Figure 7. Comparison of relative abundance of the top 10 phyla across intestinal sites and in HC and IBS-D patients. Kruskal-Wallis was used to detect the global difference, while the Wilcoxon test was used to detect the variation across intestinal sites in HC and IBS-D patients by pairwise comparisons. *: $p < 0.05$; **: $p < 0.01$; ***: $p < 0.001$; ****: $p < 0.0001$; ns: not significant. DL_HC: duodenal luminal samples collected from HC; DL_IBS-D: duodenal luminal samples collected from IBS-D patients; DM_HC: duodenal mucosal samples collected from HC; DM_IBS-D: duodenal mucosal samples collected from IBS-D patients; RL_HC: rectal luminal samples collected from HC; RL_IBS-D: rectal luminal samples collected from IBS-D patients; RM_HC: rectal mucosal samples collected from HC; RM_IBS-D: rectal mucosal samples collected from IBS-D patients.

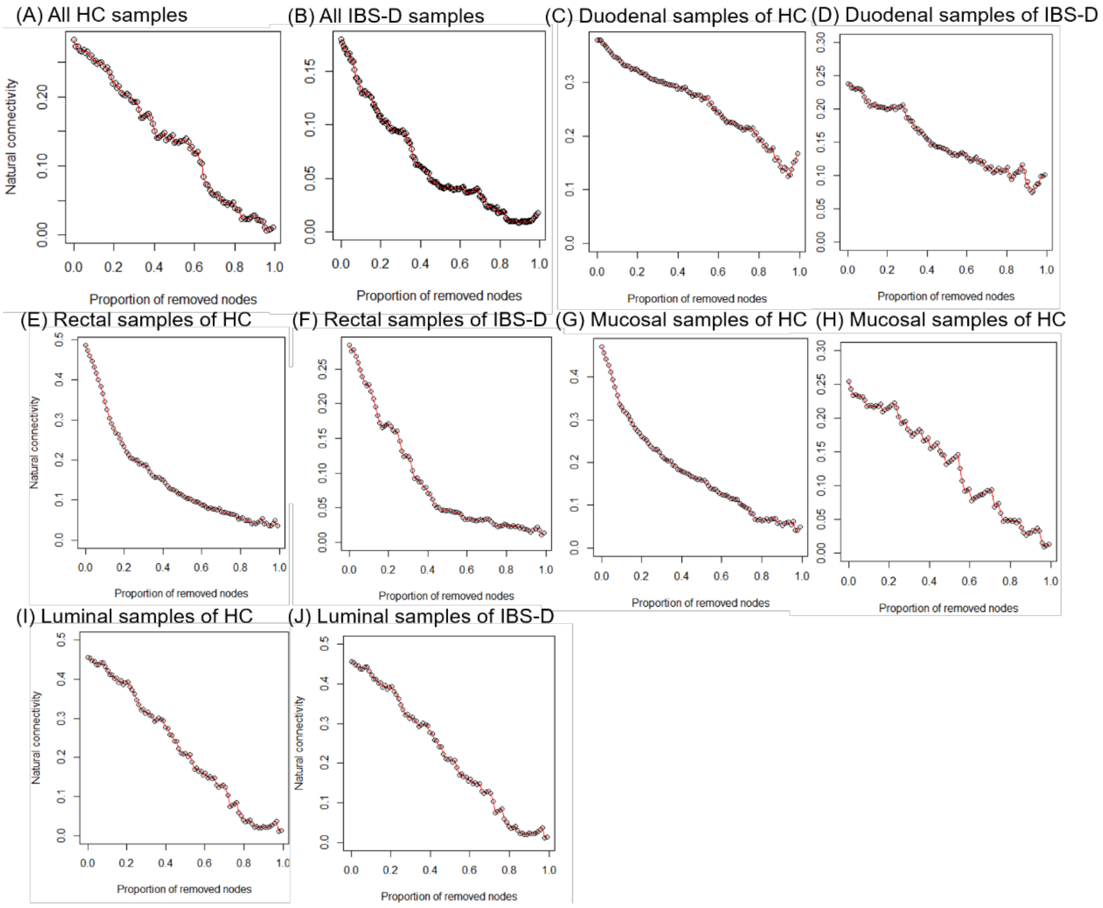

Supplementary Figure 8. Microbial co-occurrence network connectivity at different intestinal sites at genus level. Network connectivity and robustness were visualized based on (A) HC samples, (B) IBS-D samples, (C) duodenal samples collected from HC, (D) duodenal samples collected from IBS-D, (E) rectal samples collected from HC, (F) rectal samples collected from IBS-D, (G) mucosal samples collected from HC, (H) mucosal samples collected from IBS-D, (I) luminal samples collected from HC, and (J) luminal samples collected from IBS-D. The x-axis means the proportion of removed nodes (microbes), and the y-axis represents the natural connectivity of the microbial co-abundant network. The microbial co-abundant network connectivity was decreased as the nodes were removed.

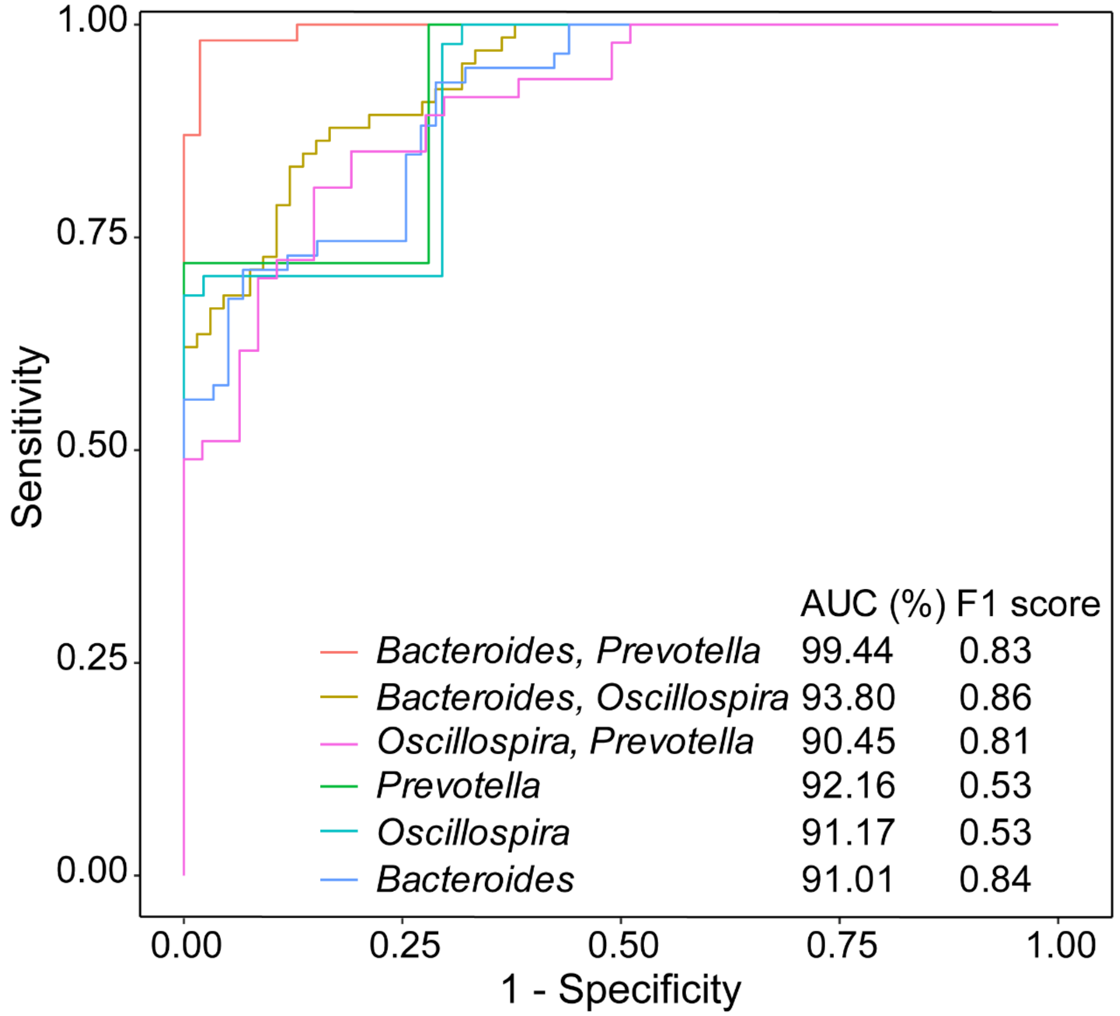

Supplementary Figure 9. Comparing the performance of sub-combinations of *Bacteroides*, *Prevotella* and *Oscillospira* in discriminating IBS-D from HC. The prediction power based on RM-specific biomarkers (*Bacteroides*, *Prevotella* and *Oscillospira*) was assessed by F1 score and AUC measures. HC: healthy controls; IBS-D: diarrhea-predominant irritable bowel syndrome. RM: rectal mucosa. AUC: area under receiver operating curve.

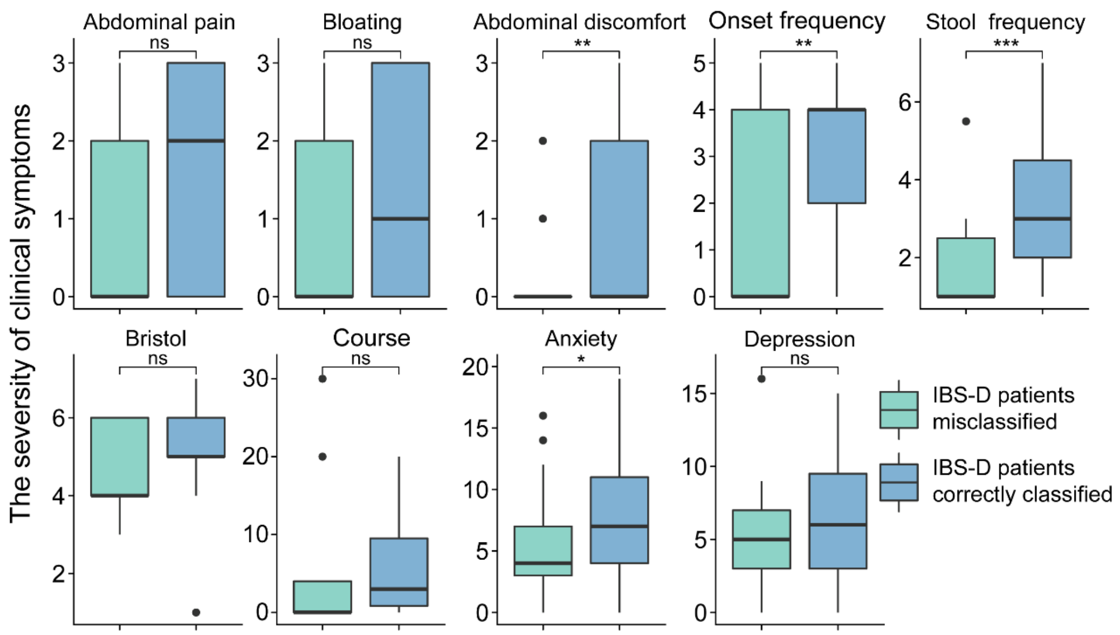

Supplementary Figure 10. IBS-D patients misclassified by RF model using RM site-specific have weaker clinical symptoms compared with patients that classified correctly. The abdominal pain, bloating, abdominal discomfort, onset frequency, stool frequency, stool consistency, course, anxiety and depression of misclassified IBS-D patients was different from that of correctly classified IBS-D patients. T-test was used to detect the significant differences in clinical symptoms between misclassified and classified IBS-D patients. *: p < 0.1. **: p<0.05; ***: p < 0.01.

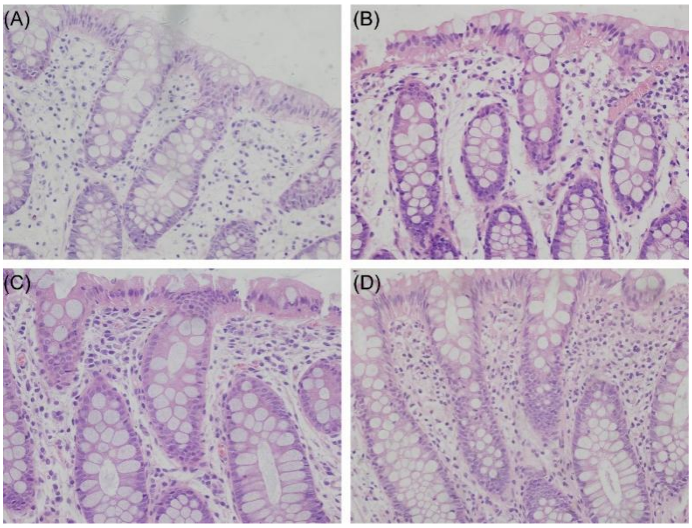

Supplementary Figure 11. HE staining for (A) duodenal mucosa in HC, (B) rectal mucosa in HC, (C) duodenal mucosa in IBS-D and (D) rectal mucosa in IBS-D has shown no inflammatory infiltration under the microscope. HC: healthy controls; IBS-D: diarrhea-predominant irritable bowel syndrome.

Supplementary Table 1. Clinical symptoms for IBS-D patients and healthy controls (HC).

**Clinical symptoms**	**HC (n= 20)**	**IBS-D (n = 74)**	**p value**
Age (years)	35.89 ± 10.49	37.34 ± 11.09	0.611
Gender (F/M)	12/7	48/25	0.832
BMI	21.53 ± 2.25	21.54 ± 3.44	0.993
Abdominal pain	NA	1.65 ± 1.22	NA
Bloating	NA	1.82 ± 1.13	NA
Abdominal discomfort	NA	0.97 ± 1.18	NA
Course (years)	NA	7.54 ± 7.43	NA
Onset frequency	NA	3.97 ± 0.99	NA
Frequency of defecation (per day)	1.11 ± 0.27	3.69 ± 1.32	<0.001
Stool consistency (BSF)	3.84 ± 0.83	5.57 ± 0.7	<0.001
HAD anxiety	3.53 ± 2.44)	8.71± 4.68	<0.001
HAD depression	3.47 ± 2.34	6.88 ± 3.79	<0.001

Note: All the data in the table was shown as mean ± SD (stand deviation). Chi-squared test was used to detect the gender distribution of all participants. T-test was used to compare the difference of other clinical symptoms between HC and IBS-D patients. The severity of abdominal pain, abdominal discomfort and bloating were classified into not at all, mild, moderate and severe, which were represented by numbers zero, one, two and three, respectively. BMI: body mass index. BSF: bristol stool form. HAD: hospital anxiety and depression scale. NA: not available. HC: healthy controls; IBS-D: diarrhea-predominant irritable bowel syndrome.

Supplementary Table 2. Comparison of the top ten genera across hosts and intestinal sites.

	HC vs. IBS-D				DM vs. RM		DM vs. DL		DL vs. RL		RM vs. RL	
	DM	DL	RM	RL	HC	IBS-D	HC	IBS-D	HC	IBS-D	HC	IBS-D
Sutterella	**	*	ns	**	***	***	ns	ns	***	***	ns	ns
Fusohacterim	ns	ns	ns	ns	ns	ns	***	***	***	***	ns	ns
Dialister	***	ns	***	**	ns	**	ns	*	ns	ns	ns	ns
Ochrobactrm	ns	***	ns	***	ns	ns	ns	ns	***	***	***	***
Lactobacillus	ns	ns	ns	ns	ns	ns	ns	ns	ns	***	ns	***
Faecalibacterium	**	***	***	*	***	***	ns	ns	***	***	ns	ns
Prevotella	ns	*	ns	*	ns	ns	ns	ns	ns	***	ns	***
Bacillus	***	**	*	**	ns	***	ns	***	***	***	***	***
Streptococcus	ns	ns	ns	ns	ns	***	**	***	***	***	ns	ns
Bacteroides	ns	***	ns	ns	***	***	ns	*	***	***	ns	ns
Pseudomonas	ns	ns	ns	ns	**	ns	ns	ns	***	***	ns	***
Acinetobacter	ns	ns	ns	***	**	ns	**	ns	**	***	**	***
Burkholderia	***	***	**	ns	ns	ns	ns	ns	***	ns	ns	*

Note: Wilcoxon test was used to detect the variation of genera across intestinal sites and hosts. *: $p < 0.1$; **: $p < 0.05$; ***: $p < 0.001$; ns: not significant. The p value was adjusted by Benjamini and Hochberg (BH) methods. HC: healthy controls; IBS-D: diarrhea-predominant irritable bowel syndrome; DM: duodenal mucosa; DL: duodenal lumen; RM: rectal mucosa; RL: rectal lumen.

Supplementary Table 3. IBS-D patients misclassified as HC in RM model could be identified as IBS-D correctly in the DL model.

Patient	Status	RM SampleID	*Prevotella*	*Oscillospira*	*Bacteroides*	RM model result	DL SampleID	*Porphyromonas*	*Sphingomonas*	*Veillonella*	*Bulleidia*	*Leptotrichia*	*Rothia*	DL model result
SISDS16	IBS-D	SISDS16RM	0.0211	0.0133	0.4079	HC	SISDS16DL	0.0000	0.0069	0.0022	0.0000	0.0000	0.0000	IBS-D
SISDS03	IBS-D	SISDS03RM	0.1101	0.0126	0.1041	HC	SISDS03DL	0.0000	0.0078	0.0014	0.0000	0.0000	0.0000	IBS-D
SISDS19	IBS-D	SISDS19RM	0.3492	0.0073	0.0608	HC	SISDS19DL	0.0034	0.0083	0.0192	0.0058	0.0192	0.0030	IBS-D
SISDS07	IBS-D	SISDS07RM	0.3774	0.0017	0.0897	HC	SISDS07DL	0.0215	0.0029	0.0430	0.0091	0.0120	0.0044	IBS-D
IBSD1001	IBS-D	IBSD1001RM	0.0013	0.0000	0.2526	HC	IBSD1001DL	0.0013	0.0023	0.0278	0.1670	0.0000	0.0000	IBS-D
IBSD0801	IBS-D	IBSD0801RM	0.1750	0.0149	0.1837	HC	IBSD0801DL	0.0808	0.0029	0.0273	0.0000	0.0062	0.0066	IBS-D
IBSD1501	IBS-D	IBSD1501RM	0.5341	0.0049	0.0579	HC	IBSD1501DL	0.0035	0.0028	0.0152	0.0094	0.0010	0.0000	IBS-D

The RM model refers to the RF model built using the combination of *Bacteroides*, *Prevotella* and *Oscillospira* in RM, while the DL model represents the RF model built using the combination of *Sphingomonas*, *Porphyromonas*, *Rothia*, *Leptotrichia*, *Bulleidia* and *Veillonella* in DL. RF: random forest; RM: rectal mucosa; DL: duodenal lumen.

Supplementary Table 4. The number of samples collected from four representative intestinal sites of 20 healthy individuals and 74 IBS-D patients.

	Duodenal mucosa (DM)	Duodenal lumen (DL)	Rectal mucosa (RM)	Rectal lumen (RL)
**HC**	20	19	20	17
**IBS-D**	37	43	53	74

Note: HC: healthy controls; IBS-D: diarrhea-predominant irritable bowel syndrome.
